# Supplementary material for: Physiological and Biochemical Responses and Transcriptome Analysis of Bangia fuscopurpurea (Rhodophyta) Under High-Temperature Stress
Source: Curr Issues Mol Biol. 2025 Jun 25;47(7):484. doi: 10.3390/cimb47070484 (PMC12293457; doi:10.3390/cimb47070484)
Supplement: Supplementary file 1 [file cimb-47-00484-s001.zip › Supplementary material.pdf]

(A)

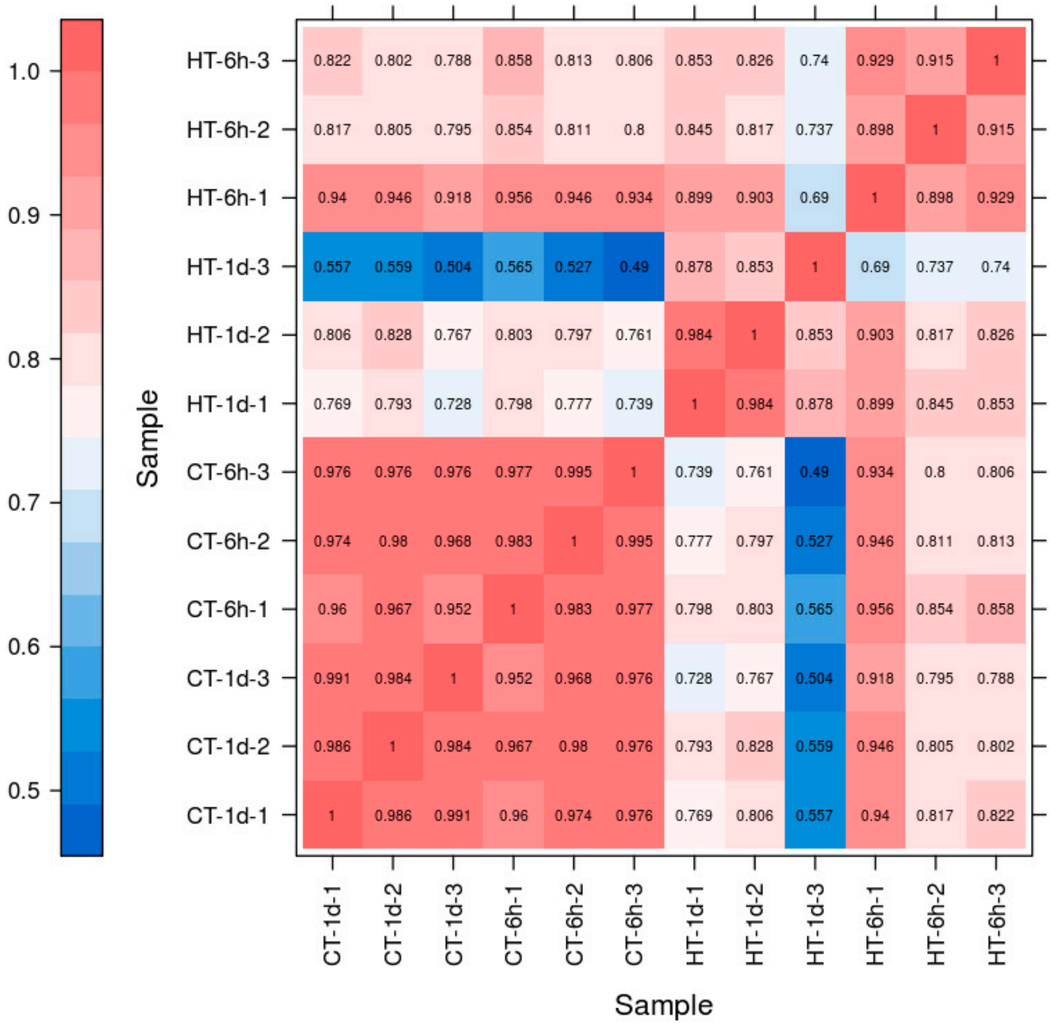
1.0
0.9
0.8
0.7
0.6
0.5
Sample
HT-6h-3 0.822 0.802 0.788 0.858 0.813 0.806 0.853 0.826 0.74 0.929 0.915 1
HT-6h-2 0.817 0.805 0.795 0.854 0.811 0.8 0.845 0.817 0.737 0.898 1 0.915
HT-6h-1 0.94 0.946 0.918 0.956 0.946 0.934 0.899 0.903 0.69 1 0.898 0.929
HT-1d-3 0.557 0.559 0.504 0.565 0.527 0.49 0.878 0.853 1 0.69 0.737 0.74
HT-1d-2 0.806 0.828 0.767 0.803 0.797 0.761 0.984 1 0.853 0.903 0.817 0.826
HT-1d-1 0.769 0.793 0.728 0.798 0.777 0.739 1 0.984 0.878 0.899 0.845 0.853
CT-6h-3 0.976 0.976 0.976 0.977 0.995 1 0.739 0.761 0.49 0.934 0.8 0.806
CT-6h-2 0.974 0.98 0.968 0.983 1 0.995 0.777 0.797 0.527 0.946 0.811 0.813
CT-6h-1 0.96 0.967 0.952 1 0.983 0.977 0.798 0.803 0.565 0.956 0.854 0.858
CT-1d-3 0.991 0.984 1 0.952 0.968 0.976 0.728 0.767 0.504 0.918 0.795 0.788
CT-1d-2 0.986 1 0.984 0.967 0.98 0.976 0.793 0.828 0.559 0.946 0.805 0.802
CT-1d-1 1 0.986 0.991 0.96 0.974 0.976 0.769 0.806 0.557 0.94 0.817 0.822
CT-1d-1 CT-1d-2 CT-1d-3 CT-6h-1 CT-6h-2 CT-6h-3 HT-1d-1 HT-1d-2 HT-1d-3 HT-6h-1 HT-6h-2 HT-6h-3
Sample

(B)

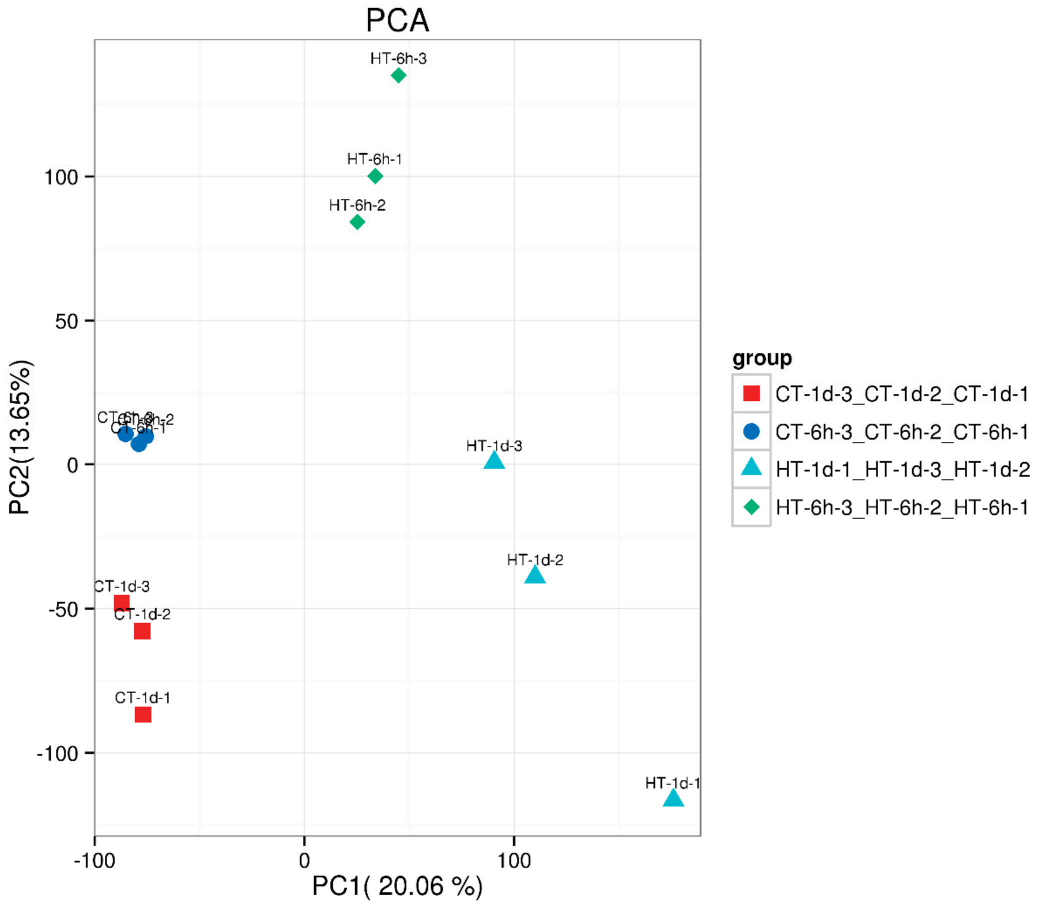

Figure S1. Sample correlation analysis. (A) Heat map of Pearson correlation coefficients. (B) Two-dimensional diagram of principal component analysis

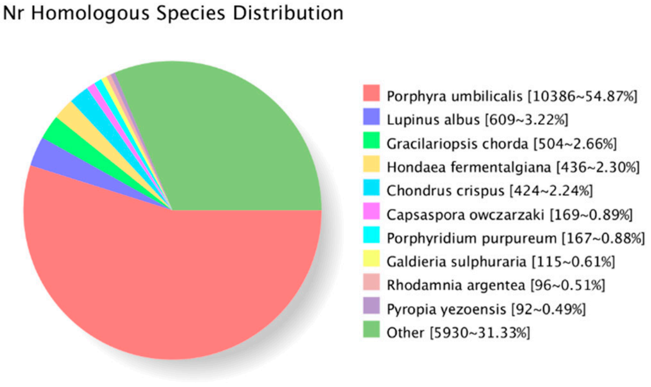

Figure S2. Statistics of the number of species compared from the Nr database based on the transcriptome results of *Bangia fuscopurpurea*.

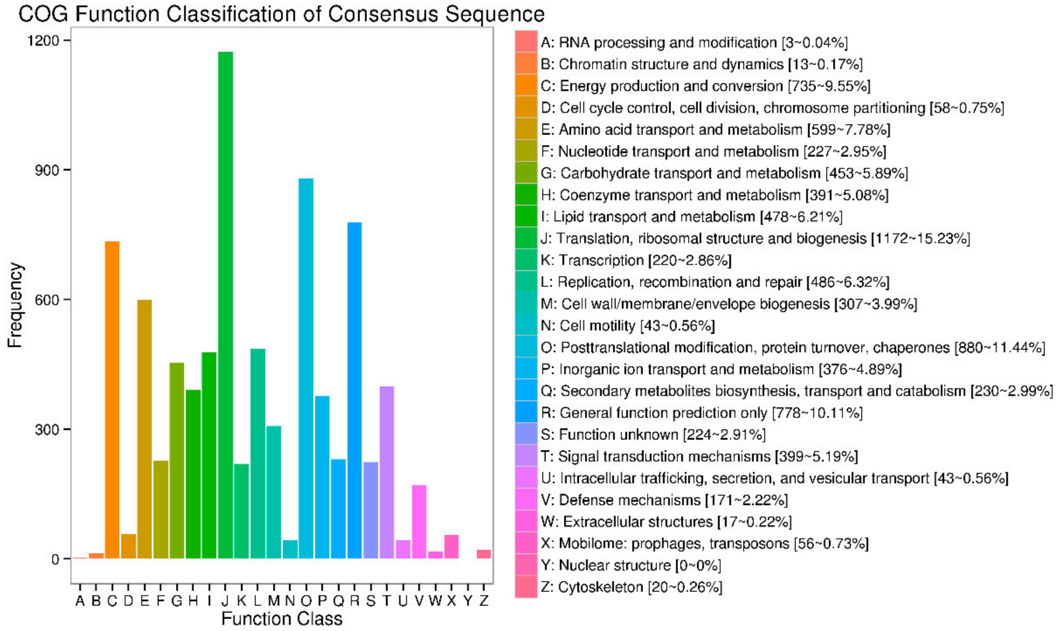

Figure S3. Statistics of COG functional classification results annotated from the COG/KOG database based on the transcriptome results of *Bangia fuscopurpurea*.

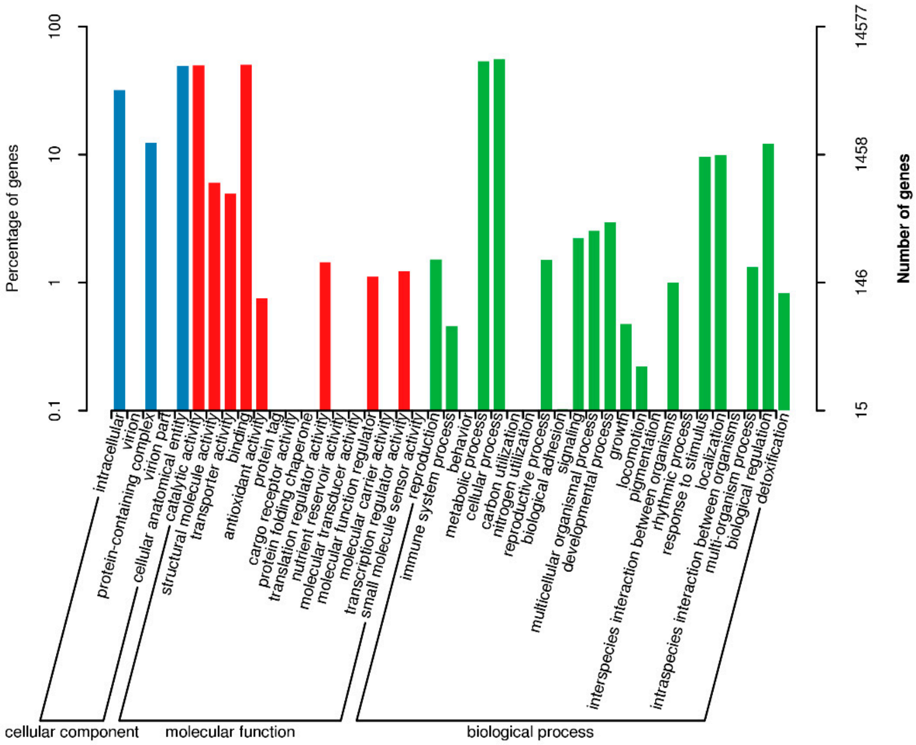

Figure S4. Gene Ontology (GO) Level 2 Classification Statistics for the transcriptome of *Bangia fuscopurpurea*.

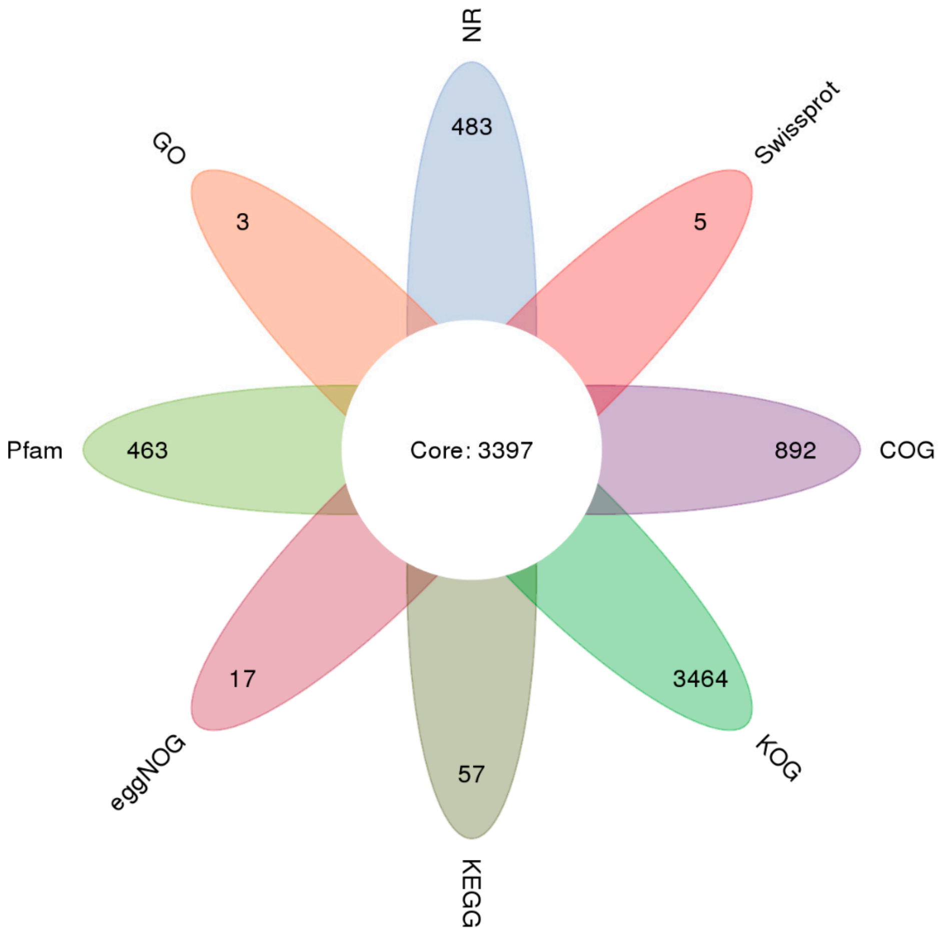

Figure S5. Four major databases annotated with flower diagrams for the transcriptome of *Bangia fuscopurpurea*.

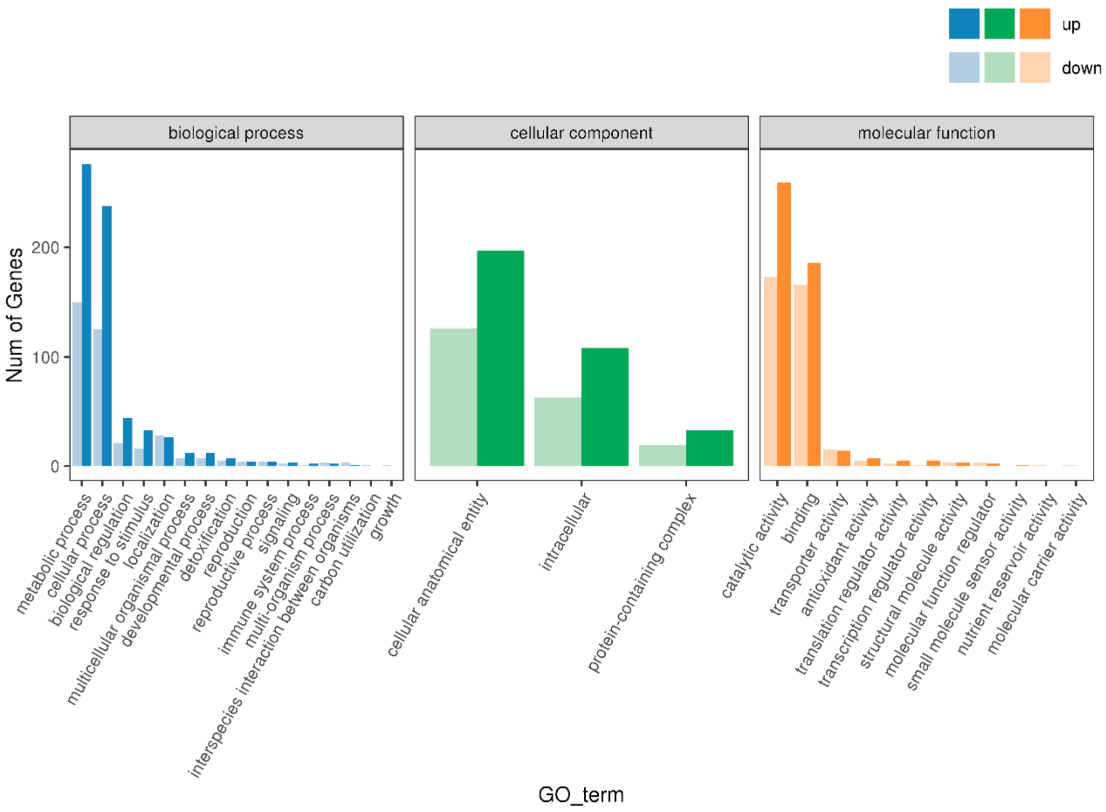

CT-6hvsHT-6h

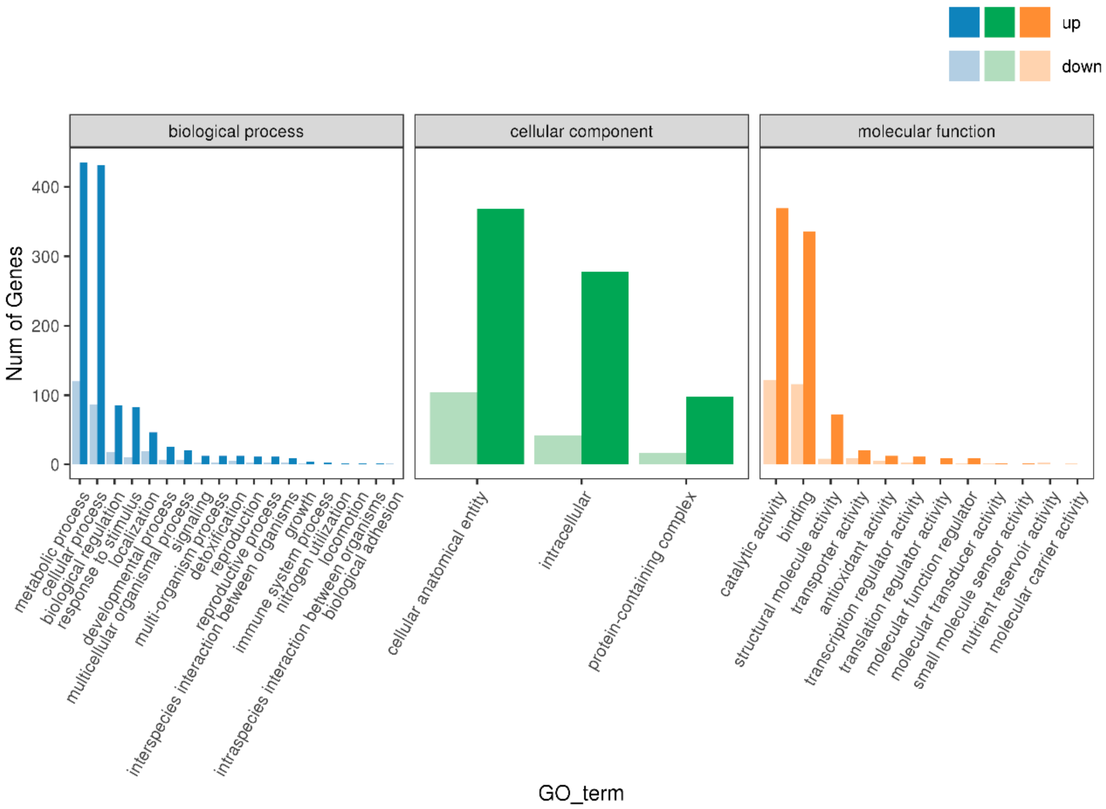

CT-1dvsHT-1d

Figure S6. GO classification of DEGs in CT-6h vs HT-6h and CT-1d vs HT-1d.

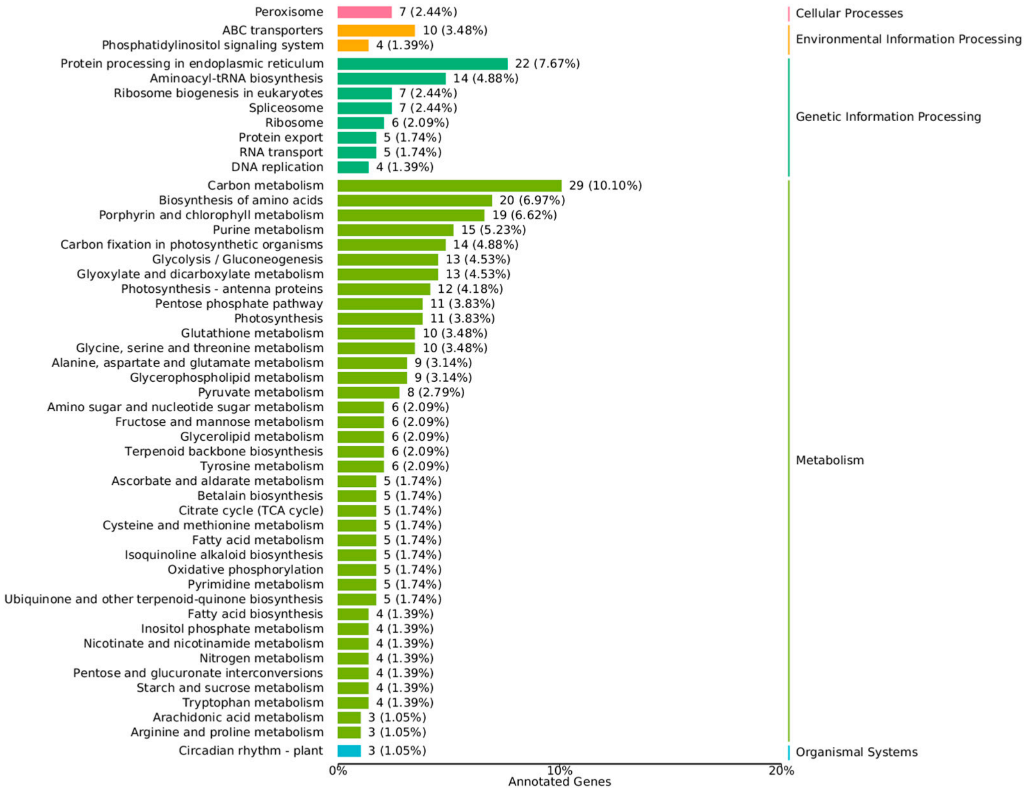

CT-6hvsHT-6h

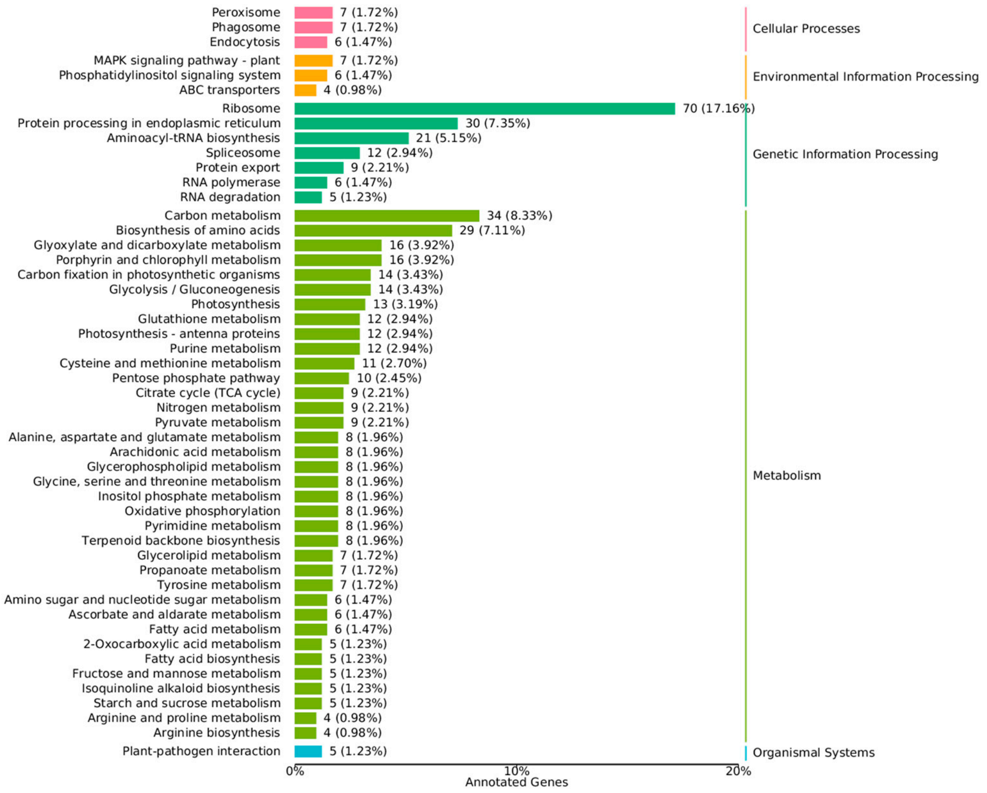

CT-1dvsHT-1d

Figure S7. KEGG classification of DEGs in CT-6h vs HT-6h and CT-1d vs HT-1d.
